# Hiccups during general anesthesia with remimazolam

**DOI:** 10.1186/s40981-024-00727-y

**Published:** 2024-09-04

**Authors:** Koh Mizutani, Masahiko Tsuchiya

**Affiliations:** 1 Department of Anesthesia, Chibune Hospital, 3-2-39 Fukumachi, Nishiyodogawa-Ku, Osaka, 555-0034 Japan; 2 Department of Anesthesia, Kishiwada Tokushukai Hospital, 4-27-1, Kamoricho, Kishiwada, 596-0042 Japan

To the editor:

We read with interest the reports of hiccups during general anesthesia that were suspected to be caused by remimazolam [1]. Decades ago, we often encountered hiccups during general anesthesia. However, they have become less frequently observed. In retrospect, this change coincided with the introduction of remifentanil. Before remifentanil, the only analgesic administered during general anesthesia was intravenous fentanyl or epidural anesthesia. These agents likely did not inhibit the diaphragmatic stimulation causing hiccups during abdominal surgery or the associated reflex arcs originating in the brainstem [2]. On the other hand, remifentanil’s analgesic effect appears sufficient to inhibit both. In the present case, hiccups occurred despite a sufficient dose of remifentanil and bispectral index (BIS) values between 49 and 60. The reason may be that the hypnosis and reflex inhibition induced by remimazolam, a benzodiazepine, is less profound than that achieved with propofol or volatile inhaled anesthetics [3]. Benzodiazepines, propofol, and volatile inhaled anesthetics act on γ-aminobutyric acid (GABA) receptors, but at different sites of action [4], so their mechanisms of action and BIS values are different [5]. In addition, anesthesia with low BIS values obtained from forebrain electroencephalography does not mean inhibition of brainstem reflexes [6]. Therefore, remimazolam, a benzodiazepine, may not inhibit the hiccup reflex, even though the patient had sufficiently low BIS values. Although the authors concluded that remimazolam may induce hiccups, it is important to note that its weak hypnotic potency and weak reflex inhibition may also play a role.

Since hiccups are unpredictable and infrequent, randomized prospective studies are impractical. Thus, most reports on hiccups during anesthesia are case reports, making it difficult to provide evidence-based conclusions. Consequently, most reports and this letter are based solely on the subjective experiences of their authors.

## Data Availability

Not applicable.
